# Upper-limb physical activity after distal radius fractures treated operatively or nonoperatively: a secondary analysis of a multicenter randomized trial

**DOI:** 10.2340/17453674.2026.46299

**Published:** 2026-07-02

**Authors:** Lisa U TØNNING, Teemu P HEVONKORPI, Antti P LAUNONEN, Aleksi REITO, Mette S SKJÆRBÆK, Toni LUOKKALA, Helle K ØSTERGAARD, Minna K LAITINEN, Bernd GRIMM, Inger MECHLENBURG, Ville M MATTILA, the NITEP Group (Nordic Innovative Trial to Evaluate osteoPorotic fractures)

**Affiliations:** 1 Department of Orthopedic Surgery, Aarhus University Hospital, Aarhus, Denmark; 2 Department of Clinical Medicine, Aarhus University, Aarhus, Denmark; 3 Department of Orthopaedics, Tampere University Hospital, Tampere, Finland; 4 Faculty of Medicine and Health Technology, Tampere University, Tampere, Finland; 5 Department of Orthopedics, Viborg Regional Hospital, Viborg, Denmark; 6 Department of Hand Surgery, Central Finland Hospital Nova, Jyväskylä, Finland; 7 Department of Orthopaedics, Helsinki University Hospital, Helsinki, Finland; 8 Luxembourg Institute of Health, Luxembourg

## Abstract

**Background and purpose:**

While most patients recover well from distal radius fracture, some experience prolonged deficits. We aimed to primarily compare high-intensity upper-limb activity 12 months after operative vs nonoperative treatment of malaligned distal radius fractures and secondarily to assess the impact of fracture alignment and compare activity levels with healthy older adults.

This is a secondary analysis of the Distal Radius Fracture Trial.

**Methods:**

The Distal Radius Fracture Trial was a multicenter, randomized controlled trial. Patients with malaligned distal radius fractures were randomized to operative treatment with a volar locking plate or nonoperative treatment. Patients with well-aligned distal radius fractures constituted a separate non-randomized group. High-intensity upper-limb activity was measured using wrist-worn accelerometers at 3 and 12 months. Healthy subjects were recruited for comparison. Longitudinal analyses were performed using linear mixed models.

**Results:**

157 patients and 59 healthy controls provided valid accelerometer data. At 12 months patients with malaligned fractures treated operatively had significantly higher high-intensity activity in the affected upper limb than those treated nonoperatively (37 vs 28 min/day; adjusted difference 8.5 min/day, 95% confidence interval [CI] 0.3–17). Fracture alignment was not significantly associated with activity. Compared with healthy subjects, operatively treated patients showed no clear difference (2.7 min/day, CI –8.3 to 14), whereas nonoperatively treated patients demonstrated lower activity levels (12 min/day, CI 4.4–20).

**Conclusion:**

In patients with malaligned distal radius fractures, operative treatment was associated with higher high-intensity upper-limb activity at 12 months than nonoperative treatment. The activity level in operatively treated patients was similar to that observed in healthy older adults.

Since the early 2000s, the 2 main treatments for distal radius fracture have been nonoperative cast immobilization and operative fixation with a volar locking plate [1-3]. Previous meta-analyses indicate that most patients, ranging from 65% to 96%, recover well and achieve near-normal wrist function within a year, though some experience prolonged pain or functional deficits [4-7].

Objective methods are important to complement patient-reported outcome measures (PROMs), which are widely used to assess subjective recovery after distal radius fracture [8-10]. Wearable technologies offer valid measures of physical activity, although evidence for upper-limb activity measurement in orthopedic populations remains limited [11]. Wrist-worn wearables such as accelerometers can measure arm use, which is relevant after injury or surgery. PROMs are subject to issues such as floor/ceiling effects, nonlinearity, low face validity, and lack of information on the dominant side.

The Distal Radius Fracture Trial compared nonoperative treatment with operative treatment with a volar locking plate fixation and showed slight functional improvement with a volar locking plate in primarily malaligned fractures, though below the minimal clinically important difference [12]. We aimed primarily to compare high-intensity upper-limb activity 12 months after operative vs nonoperative treatment of malaligned distal radius fractures using accelerometers. Secondary aims were to assess the impact of fracture alignment on high-intensity activity, and to compare activity levels with those observed in healthy subjects.

## Methods

### Trial design

This is a secondary analysis of the Distal Radius Fracture Trial; a multicenter, randomized, controlled trial. The reporting followed the “Consolidated Standards of Reporting Trials 2025” (CONSORT 2025) statement [13]. Participants with a distal radius fracture that did not maintain acceptable alignment after closed reduction (primarily malaligned fractures) were randomly assigned in a 1:1 ratio to undergo either nonoperative treatment or operative treatment with a volar locking plate. Patients with acceptable alignment after closed reduction were followed up for potential early malalignment. If the alignment was lost at 5–10 days of follow-up, the patients were randomized in a 1:1 ratio to continue nonoperative treatment or to have operative treatment with a volar locking plate. Patients with acceptable alignment after closed reduction who maintained the alignment at 5–10 days of follow-up continued the standard follow-up visits for nonoperative treatment. These patients comprised the well-aligned distal radius fractures group.

### Patients and healthy subjects

The study has 3 samples of participants: (i) patients with a malaligned distal radius fracture (randomized to nonoperative or operative treatment), (ii) patients with a well-aligned distal radius fracture, and (iii) healthy subjects. The 2 samples of patients were 65 years or older with a distal radius fracture and recruited from Tampere University Hospital (Finland), Central Finland Hospital Nova (Finland), and Viborg Regional Hospital (Denmark). The patients included in this analysis represent a subsample of the Distal Radius Fracture Trial—specifically those with available activity measures, as not all participating centers in the Distal Radius Fracture Trial collected activity data. The inclusion criteria followed the Distal Radius Fracture Trial protocol: low-energy intra- or extra-articular dorsally displaced distal radius fracture (AO classification type A or C within 3 cm of the radiocarpal joint, diagnosed with lateral and posterior–anterior radiographs. The radiographic inclusion criteria were: more than 10° dorsal tilt and/or more than 2 mm step-off and/or more than 3 mm shortening in radiographs. The exclusion criteria of the trial were refusal to participate in the study, open fracture more than Gustilo Grade 1, Chauffeur’s, Barton’s, or Smith’s fracture, inability to understand written and spoken guidance in local languages, and pathological fracture or previous fracture in the same wrist or forearm.

Patients treated operatively underwent fixation with a volar locking plate. A modified Henry’s approach was used and the wound was closed with absorbable sutures. A dorsal functional position cast was applied for 2 weeks following surgery. Patients treated nonoperatively received a dorsal functional cast for 5 weeks. Following cast removal, all patients underwent an exercise program guided by a physiotherapist or an occupational therapist. The exercise program has been described previously [12].

Healthy subjects aged 65 years or older were recruited face-to-face by research personnel at community centers in Aarhus, Denmark, and each provided written informed consent prior to participating. These community centers are publicly accessible facilities offering social, recreational, and activity-based programs for older adults living independently in the community. The research personnel had a stand at the community centers and asked potential subjects who used the community center to participate. The inclusion criteria were: age 65 years or older, no previous fractures in the hand, arm, or shoulder, the ability to wear the wristband with the accelerometers for the following 4 days, and the ability to read and understand Danish. No exclusion criteria were applied, and the healthy subjects therefore constituted a convenience sample.

### Randomization and blinding

After informed consent was obtained, participants with a malaligned fracture were allocated with an online randomization system (http://randomize.net), where the researcher logged on and received the allocated intervention. Patients were randomized in a block allocation fashion. Blocks of 10 were stratified by age (65–74 years and 75 years or older), sex, and intra-articular vs extra-articular fracture. The researchers did not have access to the allocation sequence. Participants were aware of the treatment assignments in the trial. Physicians responsible for the interventions did not participate in collecting the primary outcomes during the follow-up. The researcher responsible for accelerometer data processing and statistical analyses was blinded to treatment allocation until all analyses were completed.

### Activity monitoring and procedure

Accelerometer sensors (AX3, Axivity Ltd, Newcastle, UK) were used to measure high-intensity upper-limb activity continuously (24/7) for at least 3 consecutive days, 3 and 12 months after initiation of fracture treatment. The wrist-worn accelerometers recorded acceleration in 3 dimensions at a sampling rate of 50 Hz and ±16g [11]. At 3- and 12-months’ follow-up, patients were instructed by therapists to wear the wristbands continuously on both wrists for 3 to 4 days. Data for healthy controls was collected once using the same procedure. After use, the accelerometers were returned, and data was downloaded using the OMGUI Configuration and Analysis Tool (v1.0.0.43, Axivity Ltd, Newcastle, UK).

### Data processing

The data was analyzed by the first author (LUT) by using a custom MATLAB script and algorithm (MathWorks, Natick, MA, USA) developed specifically for this purpose [14]. The data was segmented by day, and both the number and intensity of accelerations were calculated for each day as described by Lipperts et al. [15]. The algorithm categorized these accelerations into 9 levels based on gravitational force [15]. A cutoff of 0.5 g was predefined to distinguish between low- and high-intensity activity. As no established upper-limb specific threshold for high-intensity activity exists, this cutoff was chosen pragmatically based on expert input during development of the algorithm. During data processing, days with less than 10 h of recorded wear time were excluded. The remaining valid days were combined to calculate the average time spent on high-intensity activity for each participant [16].

### Statistics

A linear mixed model was used, using the same modeling and approach as used in the Distal Radius Fracture Trial, to compare high-intensity activity from 3 and 12 months after the distal radius fracture, between patients with a malaligned fracture randomized to nonoperative treatment and patients with a malaligned fracture randomized to operative treatment. The treatment group, age group (65–74 years of age or 75 years or older), intra- or extra-articularity of the fracture, and whether the patient was affected in their dominant or non-dominant upper limb, were considered the fixed effects. Patients with a malaligned fracture were also compared with patients with a well-aligned fracture, despite randomization. A similar linear mixed model was also used for this analysis, with fracture alignment and upper-limb dominance as fixed effects. Comparisons between patients with a distal radius fracture and healthy subjects were conducted using Student’s t-test, after visual assessment of data distribution using histograms and normal Q–Q plots. The average high-intensity upper-limb activity of the 2 arms was used for the healthy subjects. All statistical analysis was conducted in STATA version 19.0 (StataCorp LLC, College Station, TX, USA) and presented as means with 95% confidence interval (CI).

Missing outcome data occurred both within days of measurement and at follow-up. For days of measurement, accelerometer data was considered invalid and excluded if wear time was less than 10 h per day. Inclusion in the analysis required valid accelerometer data at the 3-month follow-up. In the analysis process, 2 days of data were excluded from a Danish patient, due to insufficient wear time. One day, from each arm, led the patient to be completely excluded, as no data was left for analysis. Further, 1 day of data from a healthy subject’s arms was excluded because it contained less than 10 h of recorded measurements. The healthy subject was not excluded, as the rest of the days contained valid data. At follow-up, missing data occurred due to non-returned accelerometers, accelerometers returned without recorded data (empty devices), or patients not attending the follow-up. All missing data was assumed to be missing at random. For this secondary analysis, we did not perform a formal power or sample size calculation, but used data obtained from the 171 participants in the Distal Radius Fracture Trial. No formal statistical analysis plan was made for this study; however, the primary endpoint and the statistical approach followed the statistical analysis plan for the primary endpoint in the Distal Radius Fracture Trial [12].

### Ethics, data sharing plan, funding, use of AI, and disclosures

The Distal Radius Fracture Trial was approved by the Regional Ethics Committee of Tampere University Hospital, Finland (ETL-code R16105 issued June 14, 2016) and the Scientific Ethics Committee of the Central Denmark Region, Denmark (case number 1-10-72-250-17 issued September 11, 2017). Research permits were obtained from the hospital districts prior to the commencement of the trial. The trial was conducted in accordance with the Declaration of Helsinki. All patients provided written informed consent to participation. The trial was registered at ClinicalTrials.gov (NCT02879656), and the trial protocol, specifying physical activity measurement as a secondary outcome, was published simultaneously with the onset of the trial [17].

Study data cannot be shared publicly because of confidentiality requirements under Finnish and Danish Data Security legislation. The de-identified patient data is available from the NITEP Group (contact via https://www.nitep.eu/) for researchers who meet the criteria to access confidential data.

AI tools (Microsoft Copilot/ChatGPT) were used for minor language editing and phrasing suggestions during manuscript preparation. AI tools did not generate scientific content, interpret data, perform analyses, or influence the study’s methodology or conclusions. All authors take full responsibility for all parts of the manuscript.

This trial was supported by the Academy of Finland, Sigrid Juselius Foundation Finland, Finnish Insurance Companies grant, Finnish State Research Funding, and Dagmar Marshall Foundation. The funders had no role in the study design, data collection and analysis, decision to publish, or manuscript preparation.

Complete disclosure of interest forms according to ICMJE are available on the article page, doi: 10.2340/17453674.2026.46299

## Results

171 patients with a distal radius fracture were enrolled across 3 study sites. After excluding patients who did not have valid accelerometer data at the 3-month follow-up, as defined a priori, 157 patients were included in the analysis, of which 64 (41%) had a malaligned fracture and received nonoperative treatment, 52 (33%) had a malaligned fracture and received operative treatment, and 41 (26%) had a well-aligned fracture and received nonoperative treatment (Figure). Additionally, 60 healthy subjects were recruited, with 59 analyzed after exclusions. Demographics are presented in Table 1.

**Figure:**
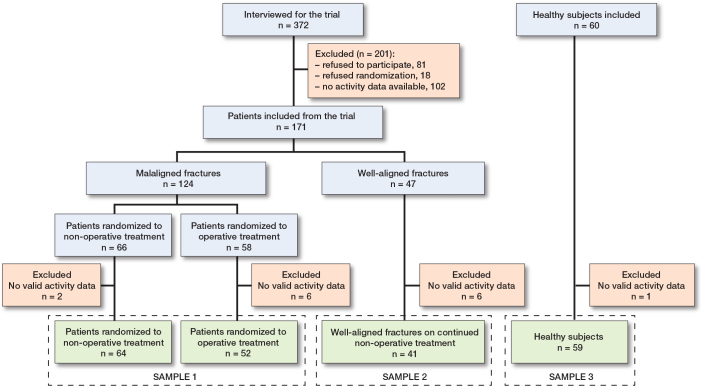
Flowchart of included patients and healthy subjects.

**Table 1:** Baseline characteristics of included patients and healthy subjects. Values are count unless otherwise specified

Factor	Sample 1 Patients with a malaligned fracture		Sample 2 Patients with a well-aligned fracture	Sample 3 Healthy subjects
	Non-operative n = 64	Operative n = 52	Non-operative n = 41	No treatment n = 59
Factor	n = 64	n = 52	n = 41	n = 59
Female sex	53	51	37	43
Age, mean (SD)	74 (5.7)	74 (5.7)	73 (6.2)	78 (7.5)
Age group				
< 75 years	36	29	26	22
≥ 75 years	28	23	15	37
Upper-limb dominance				
Right	61	50	40	53
Left	3	2	1	6
Fractured upper limb				
Right	26	26	20	
Left	38	26	21	
Fracture severity				
Intra-articular	36	27		
Extra-articular	28	25		

SD: standard deviation.

### Outcomes

In the comparison of patients with malaligned fractures, the mean high-intensity activity in the affected upper limb at 12 months was 28 min/day (Cl 22–33) for nonoperatively treated patients and 37 min/day (Cl 31–43) for operatively treated patients. The adjusted difference between these 2 groups was 8.5 min/day (CI 0.3–17), favoring the operative group (P = 0.04) (Table 2). For the non-affected upper-limb, the adjusted between-group difference at 12 months was 2 min/day (CI –7.3 to 12) for the operative vs nonoperative group (P = 0.7) (Table 2).

**Table 2:** Comparison of high-intensity activity in the affected and the non-affected upper limbs, measured at 3 and 12 months after a malaligned distal radius fracture in patients randomized to nonoperative or operative treatment. Values are mean minutes/day spent on high-intensity activity with 95% confidence intervals

Upper-limb type Follow up	Nonoperative treatment n = 64	Operative treatment n = 52	Crude difference	Adjusted ^a^ difference
Affected				
3 months	27 (21–32)	31 (25–37)	4.5 (–3.5 to 12)	3.8 (–4.1 to 12)
12 months	28 (22–33)	37 (31–43)	9.4 (1.1 to 18)	8.5 (0.3 to 17)
Non-affected				
3 months	43 (37–49)	40 (33–47)	–3.4 (–13 to 5.7)	–2.5 (–12 to 6.7)
12 months	36 (30–43)	38 (30–45)	1.1 (–8.4 to 11)	2.2 (–7.3 to 12)

a Adjusted for dominant upper limb, as well as whether the fracture was intra- or extra-articular, and the patient’s age group (< 75 years or ≥ 75 years).

The adjusted difference in high-intensity activity between patients with a well-aligned fracture and patients with a malaligned fracture, regardless of treatment, was 6.8 min/day (CI –0.7 to 14), favoring the patients with a well-aligned fracture (P = 0.08) (Table 3). For the non-affected upper limb, the adjusted difference was 5.8 min/day (CI –3.3 to 15) (P = 0.2) (Table 3).

**Table 3:** Comparison of the high-intensity activity of the affected and the non-affected upper-limbs, measured 3 and 12 months after the distal radius fracture in patients with a malaligned or a well-aligned fracture. Values are mean minutes/day spent on high-intensity activity with 95% confidence intervals

Upper-limb type Follow up	Malaligned fracture n = 116	Well-aligned fracture n = 41	Crude difference	Adjusted^a^ difference
Affected				
3 months	29 (25–33)	35 (28–41)	6.2 (–1.3 to 14)	5.1 (–2.2 to 13)
12 months	32 (28–36)	40 (33–46)	7.9 (0.2 to 16)	6.8 (–0.7 to 14)
Non-affected				
3 months	42 (37–46)	47 (39–54)	5.1 (–3.9 to 14)	5.4 (–3.5 to 14)
12 months	37 (32–42)	42 (35–50)	5.5 (–3.7 to 15)	5.8 (–3.3 to 15)

a See Table 2.

For reference, healthy subjects had an average of 40 min/day (CI 33–47) of high-intensity upper-limb activity. 12 months post-fracture, patients with malaligned fractures had 8.1 min/day (CI 0.1–16) less high-intensity activity in the affected upper limbs compared with the healthy subjects. The difference was greater in the malaligned nonoperative group (12.4 min/day, CI 4.4–20) than in the operative group (2.7 min/day, CI –8.3 to 14), the latter statistically not significant. Patients with well-aligned fractures showed no significant difference (–0.5 min/day, CI –10 to 9) compared with the healthy subjects.

## Discussion

In this secondary analysis of the Distal Radius Fracture Trial investigating patients’ high-intensity upper-limb activity after distal radius fracture, we found that patients with malaligned fractures treated operatively with a volar locking plate had 8.5 min/day (CI 0.3–17) more high-intensity upper-limb activity 12 months after the fracture compared with those treated nonoperatively. The difference between the treatment groups is aligned with the findings of the primary analysis of the Distal Radius Fracture Trial, where operatively treated patients with primarily malaligned distal radius fracture reached significant but not clinically relevant better function at 12 months than nonoperatively treated patients measured with PRWE and QuickDASH [12]. However, as no established minimal clinically important difference (MCID) exists for high-intensity activity, the clinical relevance of the observed difference remains uncertain.

Malalignment of a distal radius fracture has been reported to influence the functional outcome after fracture in younger adults [18-20]. However, elderly patients may not be influenced by a malunion. In this analysis, patients with a malaligned distal radius fracture showed lower high-intensity activity at 12 months compared with healthy older adults. The difference was most pronounced in the nonoperative group. Patients treated operatively showed activity levels closer to those observed in healthy adults without statistically significant difference. Patients with well-aligned fractures showed no clear difference compared with healthy subjects.

It has previously been reported that operative treatment of distal radius fracture may benefit patients in the early stages of recovery. Some randomized controlled trials have shown that differences in wrist function between operative and nonoperative treatment diminish within the first 12 months, while others have found persistent differences at 12-month follow-up [4-7]. In the current analysis, patients with a malaligned fracture treated operatively showed a greater increase in high-intensity activity from 3 to 12 months compared with those treated nonoperatively. These findings suggest that differences in patients’ ability to engage in high-intensity wrist activity may persist beyond the early recovery phase and could be influenced by the treatment modality, which is in line with the findings regarding the PROMs of the Distal Radius Fracture Trial [12].

Upper-limb activity can be assessed using wearable sensors that are feasible for real-world use. In contrast, advanced motion-analysis systems, despite providing detailed assessment, are mainly restricted to research settings due to limited feasibility [21]. A previous study demonstrated clinically relevant discrepancies between patient-reported outcomes and objectively measured activity [22], indicating that patient-reported measures alone may not fully reflect real-world arm use after upper-extremity trauma. This highlights the need for objective and patient-relevant outcome measures to more comprehensively assess recovery in future studies and clinical practice.

### Limitations

Baseline activity was not measured, as patients could not wear the accelerometers prior to or immediately after the distal radius fracture. However, it was assumed that the baseline levels were close to data from the healthy subjects and the non-affected upper limb as reference. No prior studies were available to define the cut-off for high-intensity activity, which was set at 0.5 g. Additionally, the clinical significance remains unknown, leaving the relevance of these results uncertain. Furthermore, no sample size calculation was performed because this was a secondary analysis, as the calculation was based on the primary analysis [12]. Finally, differences in age and sex distribution between the healthy subjects and patient groups should be considered when interpreting comparisons with healthy subjects.

### Conclusion

We showed that operative treatment of patients aged 65 years or older with volar locking plate for malaligned distal radius fracture was associated with higher objectively measured high-intensity upper-limb activity at 12 months compared with nonoperative treatment, but the clinical relevance of this difference remains uncertain. The activity level in operatively treated patients was similar to that observed in healthy subjects, whereas patients treated nonoperatively did not reach similar activity levels.

## Notes

Funding acquisition: APL, TL, MKL, IM, and VMM. Planning the study: LUT, TPH, APL, AR, MSS, TL, HKØ, MKL, BG, IM, and VMM. Supervision: TPH, APL, MSS, TL, HKØ , IM, and VMM. Data analysis: LUT and BG. Statistical analysis: LUT and AR. Writing the manuscript: LUT, TPH, APL, AR, IM, and VMM. Critically revising the manuscript: LUT, TPH, APL, AR, MSS, TL, HKØ, MKL, BG, IM and VMM

The authors would like to thank Katrine Iversen, Marlene Jessing Larsen, and Rikke Sommer Haaber for help with collecting data on healthy subjects and Trine Louise Dahl Kjeldsen for her significant effort in collecting all Danish accelerometer data in the Distal Radius Fracture Trial patients.

Handling co-editors: Paul Gerdhem and Robin Christensen

*Acta* thanks Magnus Dencker and other anonymous reviewers for help with peer review of this manuscript.
